# Extracellular vesicles in reproductive biology and disorders: a comprehensive review

**DOI:** 10.3389/fendo.2025.1550068

**Published:** 2025-06-04

**Authors:** Jinguang Wang, Dan Wang, Yuemin Zhang, Pingping Sun, Lankai Yi, Ailing Han, Wenjie Zhao, Yuhua Zhang, Huagang Ma

**Affiliations:** ^1^ Reproductive Medicine Center of Weifang People’s Hospital, Weifang, Shandong, China; ^2^ Department of Hand, Foot, and Orthopedics Surgery, Weifang People’s Hospital, Weifang, Shandong, China

**Keywords:** extracellular vesicles, sperm, oocyte, fertility, embryos, embryo implantation, reproductive disorders

## Abstract

Extracellular vesicles (EVs) facilitate intercellular communication and the conveyance of bioactive substances, including proteins, lipids, and nucleic acids. They play a significant role in various reproductive biological processes, including gametogenesis, fertilization, early embryo development, and implantation. Dysfunctional EV activity is associated with various reproductive diseases, such as polycystic ovary syndrome (PCOS), endometriosis, male infertility, and recurrent pregnancy loss (RPL). This review systematically examines and categorizes current knowledge on EV functions in reproductive biology and disorders, and their potential as diagnostic and therapeutic tools. A systematic literature search from 2000 to 2024 identified studies showing EVs’ influence on gamete maturation, fertilization, embryonic development, and implantation. They also play a role in reproductive disorders by affecting insulin resistance, androgen production, inflammation, angiogenesis, sperm quality, and maternal-fetal immune tolerance. The review concludes that EVs are integral to reproductive health, with further research needed to understand their mechanisms and clinical potential.

## 1 Introduction

Infertility has become a pressing global health concern, with modern lifestyles and environmental pollution contributing to its rapid rise (1). Intercellular communication is essential for maintaining physiological homeostasis in multicellular organisms. Disruptions in intercellular communication are increasingly recognized as a key factor in infertility (2). In addition to juxtacrine signaling through tight junctions such as gap junctions for cell-to-cell communication, cells secrete a variety of molecules, including hormones, peptides, cytokines, and growth factors, into the extracellular environment to facilitate endocrine, paracrine, and autocrine signaling (3, 4). Recently, a novel mechanism of intercellular communication has been identified, involving the secretion and internalization of extracellular vehicles (EVs) (5).

**Figure 1:**
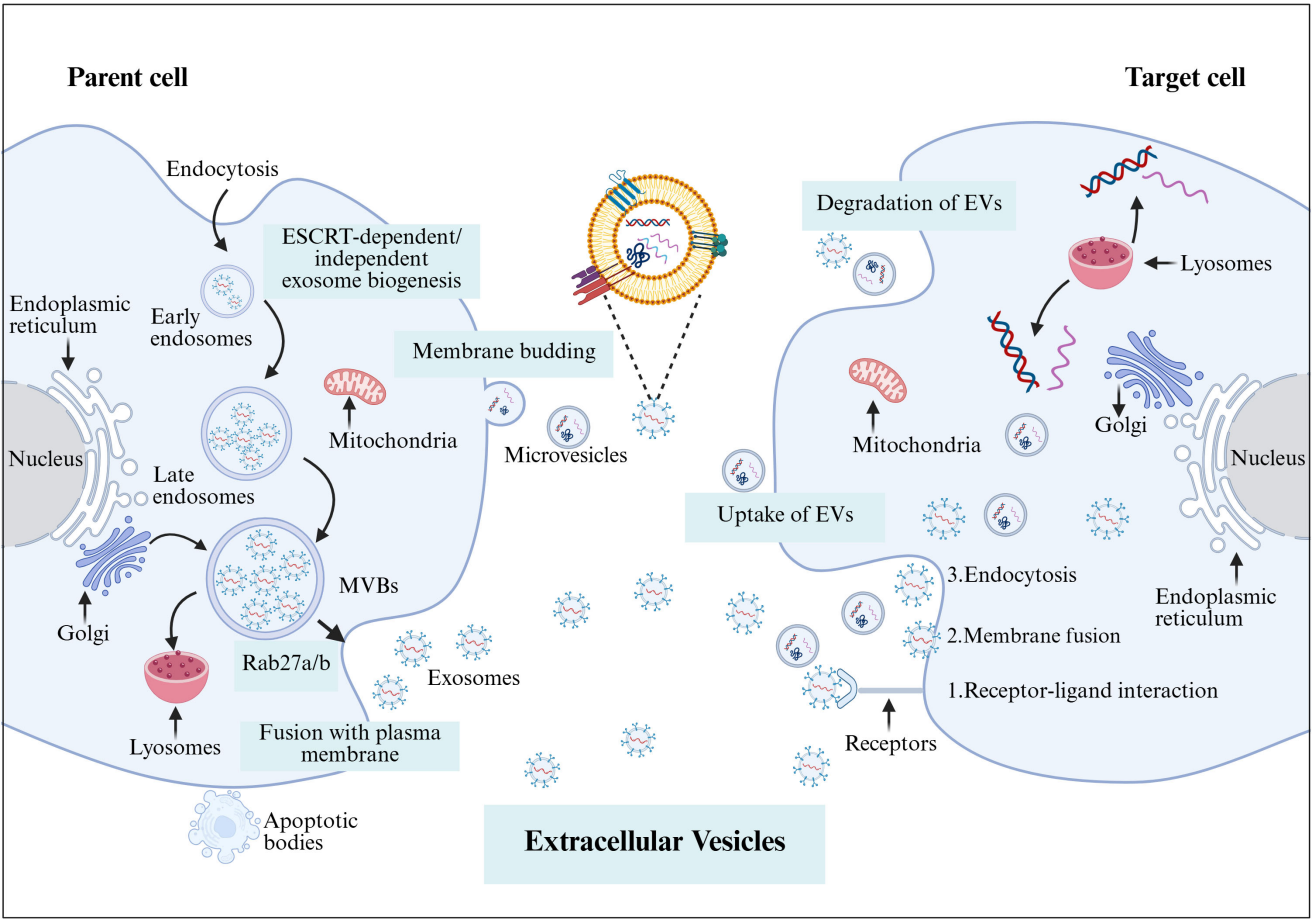
The formation of EVs begins with endocytosis, which has two pathways: returning the cargo to the plasma membrane as “recycling endosomes” or transforming into “late endosomes,” or MVBs. MVBs will either merge with the lysosome or the plasma membrane, releasing their cargo outside the cell. Several RAB proteins, including Rab 27a and Rab 27b, as well as protein complexes, help transport MVBs to the plasma membrane and release EVs. In contrast, microvesicles are formed by the plasma membrane’s outward budding and scission, whereas apoptotic cellular membranes’ outward bubbling results in the production of apoptotic bodies. EVs and target cells interact in three ways (1): membrane proteins on EVs bind directly to receptors on target cells, activating an intracellular signaling cascade; (2) EVs transport their contents to target cells by fusing with the cell membrane; and (3) EVs are engulfed by endocytosis, releasing signaling molecules. Created with BioRender.com.

**Figure 2:**
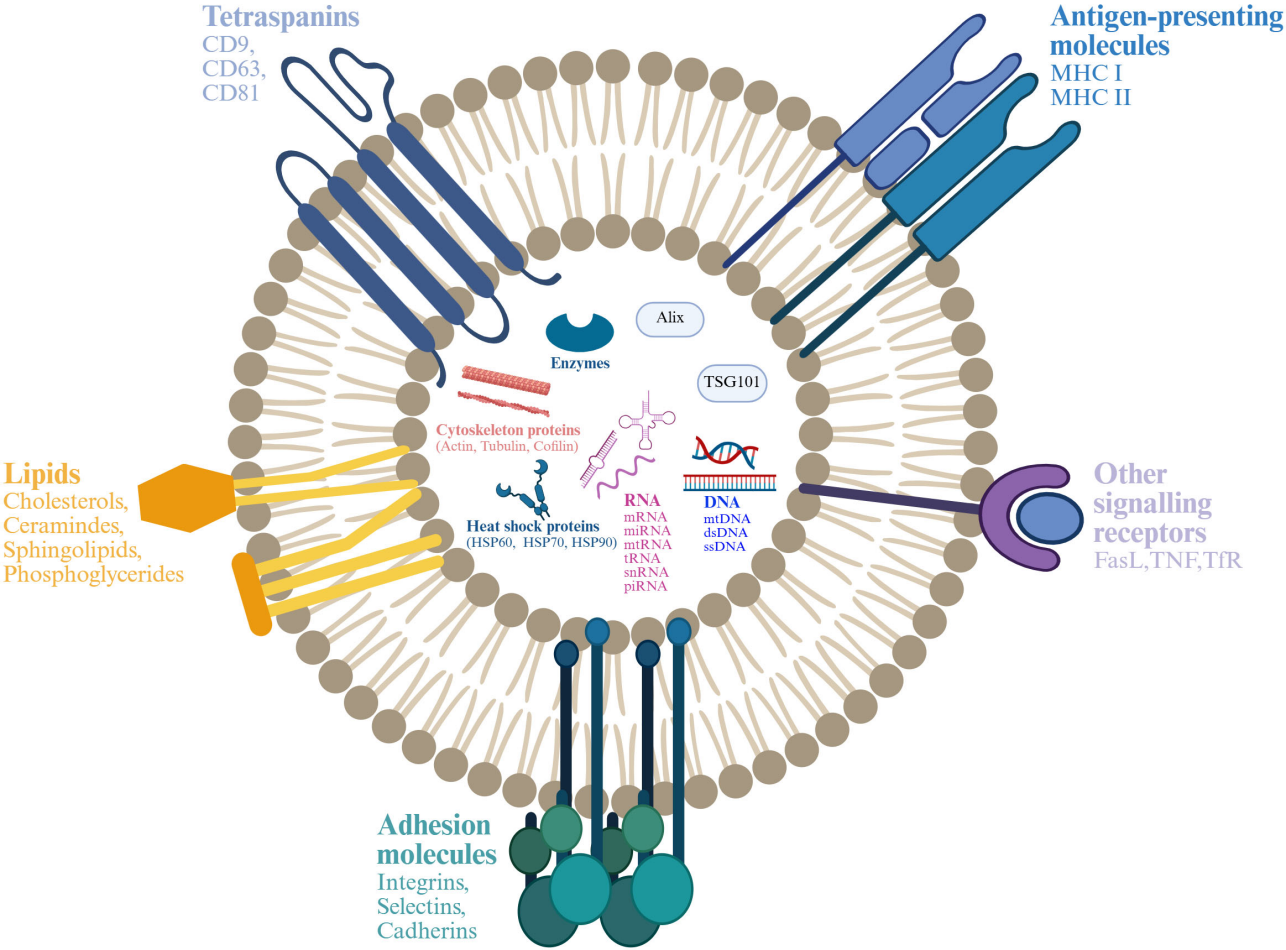
Structure and composition of EV. EV is a lipid bilayer structure that contains lipids, proteins and nucleic acids. Sphingomyelin, phosphatidylserine, cholesterol and ceramides are highly distributed on the membrane. In addition, EVs also contain a variety of proteins such as major histocompatibility complex I and II (MHC I and MHC II), proteins from the MVB machinery (ALIX, TSG101), heat shock proteins (HSP70, HSP90, HSP60), tetraspanins (CD9, CD63, CD81), receptors (FasL, TNF, TfR), adhesion molecules (Interins, Selectins, Cadherins) and cytosolic proteins, RNA and DNA. Created with BioRender.com.

EVs are divided into microvesicles, apoptotic vesicles, and exosomes based on their nature and function (6, 7). Exosomes are a separate subpopulation of EVs with diameters ranging from 30 to 150 nm and densities from 1.13 to 1.19 g/mL. They serve as vehicles for informational molecules involved in communication between cells, facilitating the transport of functional proteins and genetic information. This transport can alter the phenotype and function of recipient cells, leading to alterations in cellular fate and physiological activities (8). EVs are produced by the double invagination of the plasma membrane and the inward budding of the luminal membrane. These structures develop within the intralumenal vesicles of multivesicular bodies (MVBs), which extend inward from the luminal membrane. The formation of these structures involves mechanisms that are both endosomal sorting complexes required for transport (ESCRT) -dependent and ESCRT-independent, linking with autophagosomes and lysosomes for biomolecule degradation or plasma membrane interaction for release, thus engaging in the endocytic and transport functions of cellular materials (6) (**Figure 1**). Across physiological and pathological states, nearly all cell types, including epithelial cells, macrophages, mast cells, neurons, and mesenchymal cells, are capable of secreting EVs (9). Electron microscopy has revealed that EVs are flattened, or spherical vesicles encased in a lipid bilayer membrane, displaying a distinctive cup-like morphology (10). These EVs are widely present in several biological fluids, such as blood, urine, saliva, amniotic fluid, cerebrospinal fluid, follicular fluid (FF), and semen (11, 12). They contain a consistent set of marker proteins, specifically the tetraspannin proteins CD9, CD63, CD81, and CD82, which are currently recognized as the hallmark of EV (13). Additionally, EVs are abundant in proteins that are involved in multivesicular bodies biogenesis (such as Alix and TSG101), as well as in membrane transport and fusion (including Annexins, Flotillins, and GTPases), and heat shock proteins (for instance, Hsp60, Hsp70, and Hsp90). They also harbor significant components of the major histocompatibility complex (MHC I and MHC II) proteins, as well as a variety of lipids, including sphingomyelin, sphingosine, cholesterol, ceramide, and glycans (14–16). Additionally, EVs may encompass various types of cell surface proteins, intracellular proteins, nucleic acids, amino acids, and various metabolites (5, 17) (**Figure 2**).

EVs engage in biological activities primarily through three mechanisms. Firstly, they fuse directly with the membrane of the target cell, thereby activating downstream signal pathways; secondly, EVs are internalized by the target cell through receptor-mediated endocytosis, releasing biomolecules into the cytoplasm and subsequently activating the cell; thirdly, upon recognizing specific receptors on the target cell surface, EVs initiate signal transduction pathways in effector cells (18). Overall, the interaction between EVs and target cells facilitates intercellular communication, immune modulation, cellular differentiation, and pathological processes related to related to reproductive diseases, including polycystic ovary syndrome (PCOS), premature ovarian failure (POF), endometriosis (9) (**Figure 1B**). For the reproductive system, EVs play a multifaceted role, including gamete maturation, fertilization, embryonic development, and implantation (19). Moreover, they are associated with reproductive disorders such as PCOS (20), endometriosis (21), male infertility (22), and RPL (23). The quantity and composition of EVs are considered to be innovative biomarkers for the diagnosis and prediction of reproductive diseases (9). The aim of this review is to provide a summary of the research progress of EVs in reproductive biology, to enhance our understanding of the intercellular communication mechanisms of EVs in the reproductive system.

## 2 EVs in sperm maturation

**Figure 3:**
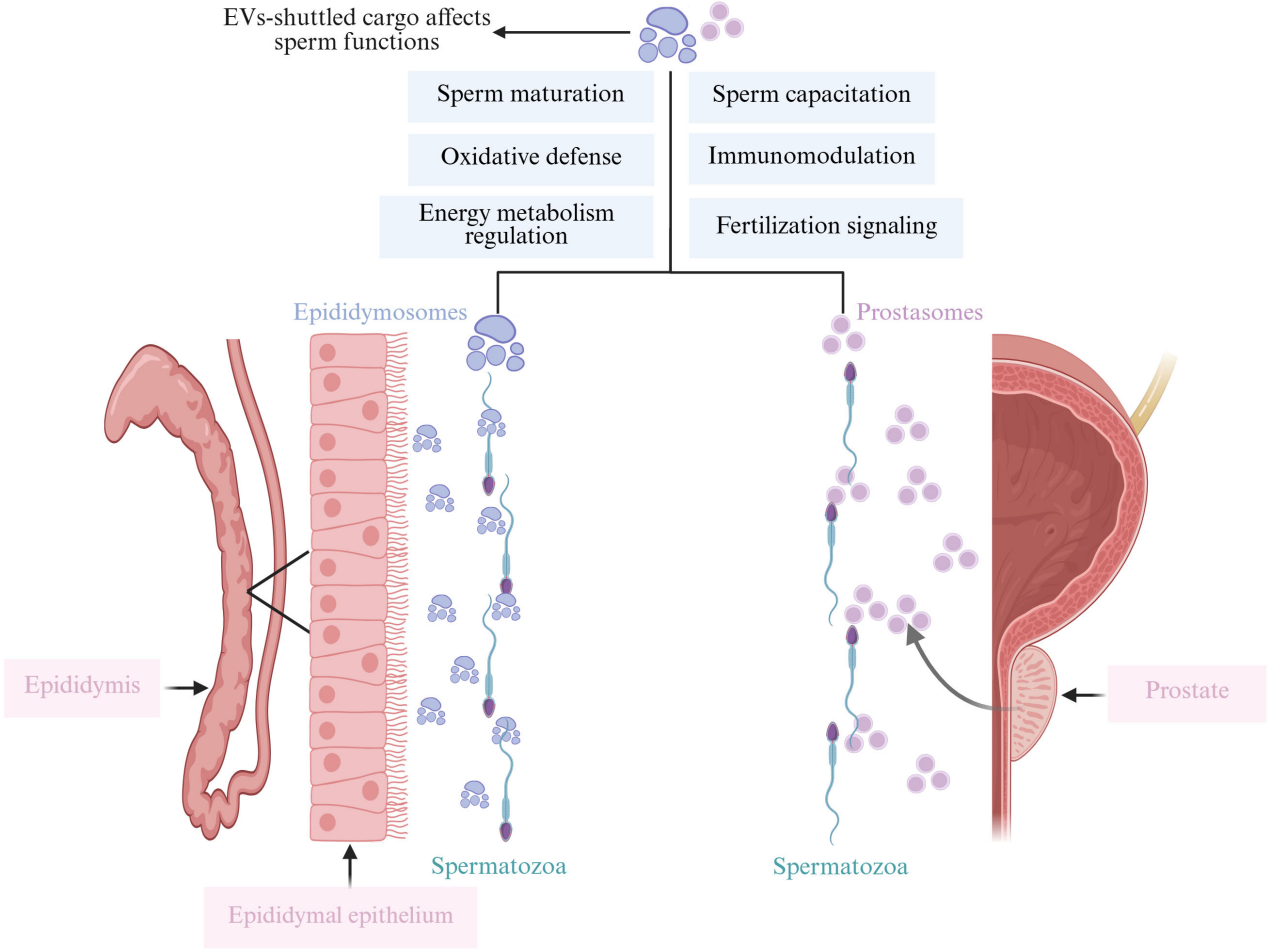
This figure illustrates how EV-shuttled cargo, released from the epididymis (epididymosomes) and the prostate (prostasomes), affects various sperm functions. Epididymosomes enhance sperm motility, maturation, mediate cell-to-cell communication, protect sperm from oxidative damage, and participate in immune regulation. Similarly, prostasomes interact with sperm to improve motility, support maturation, facilitate cell-to-cell communication, protect against oxidative damage, and modulate immune responses. Both types of EVs play crucial roles in ensuring optimal sperm function and fertility. Created with BioRender.com.

Semen is collaboratively produced by various regions of the male reproductive tract, including the testes, epididymis, vas deferens, prostate, bulbourethral glands, and other accessory glands. Comprising sperm and seminal plasma, semen plays a crucial role in reproduction as it modulates immune tolerance, facilitates sperm-egg binding, and directs pre-implantation embryonic development (24). Seminal plasma contains EVs, which constitute 3% of the total protein content and are primarily derived from the prostate and epididymis. These EVs promote sperm maturation, enhance sperm motility, and influence the tyrosine phosphorylation of sperm proteins, thereby significantly regulating the reproductive process (25) (**Figure 3**). Mammalian ejaculates contain billions of EVs, characterized by high levels of cholesterol and sphingolipids, and are laden with a variety of mRNAs and small non-coding RNAs (sncRNAs), including miRNAs, piRNAs, and siRNAs, each potentially playing regulatory roles (26).

EVs derived from the male reproductive tract play a crucial role in germ cell development and facilitate sperm maturation. Recent studies indicate that EVs secreted by Sertoli cells, containing miR-486-5p, function as communication messengers between Sertoli cells and spermatogonial stem cells (SSCs). Specifically, miR-486-5p targets and downregulates PTEN, thereby inhibiting the differentiation of SSCs (27). Furthermore, EVs secreted by Sertoli cells are capable of crossing the blood-testis barrier, supporting the viability of interstitial cells, particularly Leydig cells, which are crucial for testosterone production and overall testicular function (28). A comprehensive proteomic analysis of EVs identified a total of 2,138 proteins from semen of males diagnosed with non-obstructive azoospermia (NSP) and severe oligozoospermia (SA). Notably, 37 proteins were elevated in the NSP group, and 52 proteins increased in the SA group. This indicates that the semen’s EV proteome is closely associated with molecular processes governing sperm maturation and motility (29). In addition to proteins, microRNAs (miRNAs), Y RNAs, and tRNAs from semen also modulate sperm maturation. Sperm cytoplasmic droplets (CDs), remnants of cytoplasm, migrate distinctively in association with epididymal maturation. Sun et al. successfully isolated 348 known miRNAs and 206 novel miRNAs from EVs in porcine semen containing CDs. Compared with boar semen containing spermatozoa without CDs, 13 EV miRNAs were significantly upregulated, while 3 were notably downregulated in semen containing spermatozoa with CDs, suggesting that seminal EVs play an essential role in regulating sperm CDs (30). Moreover, extracellular adenosine triphosphate produced in boar seminal EVs modulates mitochondrial metabolism to enhance sperm motility and reduce apoptosis in fresh porcine sperm cultures (31).

Epididymosomes, also named epididymal-derived EVs, play a pivotal role in sperm growth and development. Epididymosomes, secreted by epididymal epithelial cells, contain a variety of components, including adhesive proteins such as integrins, tetraspanins, and the milk fat globule-epidermal growth factor 8 protein. These components are responsible for transferring multiple proteins to sperm, thereby promoting sperm maturation and facilitating the remodeling of the sperm membrane (32). Sperm within the epididymis undergo maturation and experience morphological and biochemical changes in an optimal microenvironment facilitated by epididymosomes (33, 34). Epididymosomes are essential for facilitating the attachment of cholesterol to the sperm membrane, enhancing sperm stability. Epididymal proteins are predominantly delivered to specific subcellular compartments or membrane domains in sperm, which are essential for acquiring fertilization capacity, regulating motility, and countering oxidative stress (26, 35). Cysteine-rich secretory protein has been shown to translocate to sperm via epididymosomes, aiding in sperm maturation (36). Epididymosomes are equipped with glutathione peroxidase, an enzyme that is vital for averting premature capacitation and safeguarding sperm against oxidative stress (37). Prostasomes, EVs derived from prostate, are believed to be significant in intercellular communication, facilitating direct interactions between fixed motile sperm and acinar cells. They contain over 140 proteins, predominantly prostate-specific enzymes, including some GPI-anchored proteins, which are crucial for sperm maturation, capacitation, and the acrosome reaction (38). It has shown that in an acidic environment, human sperm can merge with prostasomes, leveraging this interaction to transfer lipids and proteins that reduce the fluidity of the sperm membrane, thereby enhancing the reception of fertilization signals. Additionally, prostasomes, rich in cholesterol and sphingolipids, may shield sperm from the female reproductive tract’s immune responses and exhibit antioxidant and antibacterial properties (39).

It has shown that sperm co-cultured with seminal EVs at different concentrations exhibit preserved integrity, augmented total antioxidant capacity, elevated motility, and suppressed premature capacitation (40). Semen-derived EVs and their encapsulated miRNAs are correlated with male infertility. Analysis comparing EV miRNA expressions between semen samples from fertile individuals, those with obstructive azoospermia and intact spermatogenesis, and individuals with azoospermia due to spermatogenic failure, has identified significant differences in miRNA expression, specifically in miR-31-5p. Therefore, miRNA-31-5p serves as a potential biomarker for identifying azoospermia, exhibiting high sensitivity and specificity. Furthermore, miR-539-5p and miR-941 have been utilized to forecast individuals with significant spermatogenic deficiencies (41). These studies provide compelling evidence for the role of EVs in sperm maturation and identify novel avenues for the diagnosis and treatment of male infertility.

## 3 EVs in oocyte maturation

EVs are also crucial for oocyte maturation. It has shown that EVs derived from bovine follicles facilitate oocyte development by promoting cumulus cell growth and enhancing oocyte competence (42). Follicular Fluid (FF) is derived from plasma components that traverse the blood-ovarian barrier and from the secretions of granulosa and theca cells. It comprises various ions, metabolites, nucleic acids, and proteins. FF is crucial for creating a favorable microenvironment for oocyte maturation, containing hormones such as follicle-stimulating hormone (FSH), luteinizing hormone (LH), growth hormone, inhibin, and estrogens, as well as androgens and cytokines like tumor necrosis factor and Fas ligand (43). Heat stress in animals can induce oocyte damage, including mitochondrial dysfunction, and elevated levels of reactive oxygen species (44). EVs from FF can protect oocytes from heat stress, enhance the cumulus cell expansion during oocyte maturation, and improve the blastocyst formation capacity of mature oocytes under *in vitro* heat stress conditions (45).

**Figure 4:**
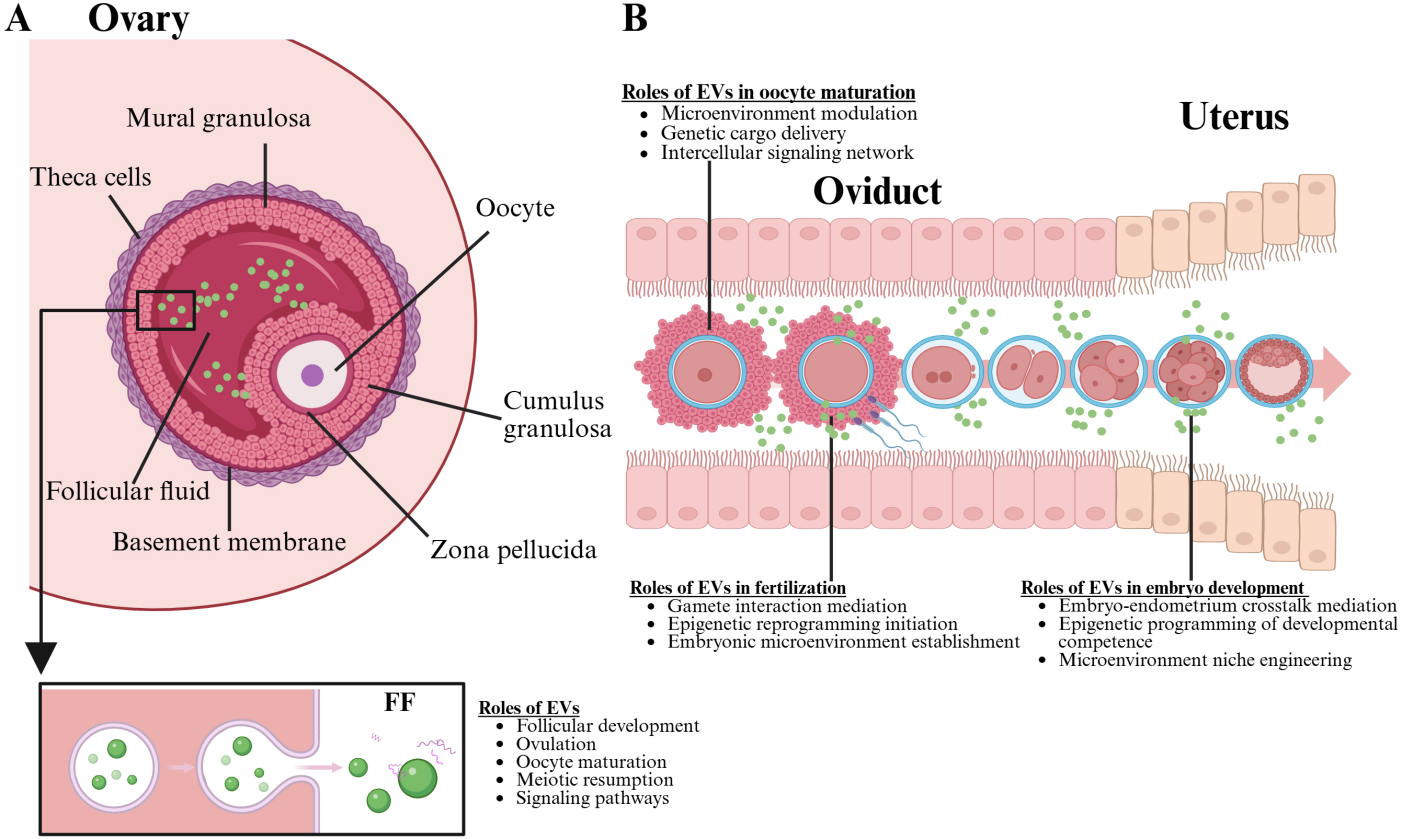
This figure illustration highlights the critical roles of EVs in the female reproductive system. Part **A**, the ovary is depicted, showcasing the formation of the oocyte and emphasizing the functions of EVs within the follicular fluid, which include promoting oocyte maturation and enhancing fertility. Part **B**, the oviduct is illustrated, detailing how EVs are essential for oocyte maturation, fertility, and early embryo development. The image underscores the multifaceted impact of EVs throughout the reproductive process. Created with BioRender.com.

Previously, it was believed that the follicle served only as a passive receiver of signals from granulosa cells. However, communication between the follicle and granulosa cells is bidirectional, involving intricate regulatory factor interactions that govern the development of both cell types. This communication can occur directly via gap junction networks, as well as through paracrine, autocrine, and endocrine regulatory mechanisms (46) (**Figure 4A**). In follicle development, the factors transmitted to the oocyte are essential for coordinating follicle development and activating various signaling molecules, including Kit, TGFB, insulin, and members of the WNT signaling family (47). In bovine early embryo development, EV miRNAs in FF are involved in regulating various signaling pathways, including ubiquitin-mediated signaling pathways, MAPK signaling pathways, insulin signaling pathways, and neurotrophic factor signaling pathways (48). A study found that FF exosomes from higher-quality oocytes show distinct miRNA profiles targeting WNT, MAPK, ErbB, and TGFβ pathways crucial for follicle development compared to low-quality oocyte FF exosomes (49). WNT proteins are secreted signaling molecules that activate Frizzled G protein-coupled receptors, which in turn facilitate follicle development, oocyte maturation, and the steroid production (50). The activation and initiation of the MAPK pathway can occur through FSH and LH, which promote the proliferation of granulosa cells and the expansion of cumulus cells (51). Conversely, the MAPK and ErbB pathways promote the resumption of oocyte meiosis by modulating cAMP levels, thereby influencing the transition from meiotic arrest to resumption (52). The intricate interactions among various factors within these pathways lead to the removal of meiosis-inhibitory factors and the activation of oocyte maturation signals.

EV-derived miR-17 and miR-92 from FF have been shown to enhance oocyte diameter and increase H4K12 acetylation levels (53). Moreover, Hu et al. identified miR-125b, let7d-5p, miR-200b, miR-26a, and miR-92a in porcine FF EVs using next-generation sequencing, suggesting their potential role in modulating porcine oocyte maturation through the TGF signaling pathway (54). EVs from bovine FF have been shown to promote granulosa cell proliferation (55). MiR-424 in EVs from FF of individuals with PCOS has been found to inhibit granulosa cell proliferation through targeting cell division cycle associated 4 (CDCA4), thereby suppressing the Rb/E2F1 signaling pathway identified to promote cell proliferation and inhibit senescence-related phenotypes (56). Similarly, EVs derived from human umbilical cord mesenchymal stem cells, enriched with miR-146a-5p or miR-21-5p, augment oocyte development in mice via the PI3K/mTOR signaling pathway, concomitantly enhancing both the quantity and quality of oocytes (57). Thus, EVs both within and outside the follicle carry various informational molecules to different cells, coordinating intercellular communication to promote oocyte development.

A series of EV miRNAs have been identified as non-invasive biomarkers for oocyte quality in assisted reproductive technologies. Notably, miR-132, miR-100, miR-99a, and miR-218 are correlated with oocyte maturation (58, 59). MiR-132, miR-212, and miR-214 may facilitate meiotic rescue by down regulation negative regulatory genes that suppress follicular maturation factors, and miR-29a may be implicated in epigenetic modifications (60). The features of miRNAs, such as miR-31a-5p, found in EVs within FF are age-dependent, potentially serving as biomarkers for the age-related decline in oocyte quality (61). Assessment of specific components within FF EVs can deepen our understanding of intra-follicular signaling and potentially uncover biomarkers for patients undergoing assisted reproductive technology treatments.

## 4 EVs in fertility

Fertilization is a multifaceted process involving oocyte stimulation, sperm binding to the zona pellucida, gamete fusion, and pronuclei development. Prostatic-like vesicles, such as prostasomes, have been shown to influence sperm function *in vitro* by inducing the acrosome reaction through their transfer to the sperm membrane (62). The inner acrosomal membrane adheres to the microvilli-rich regions of the oocyte, facilitating membrane fusion between the sperm and oocyte. Following this fusion, the oocyte membrane becomes depolarized, triggering the release of subcortical granules, which prevents polyspermy by modifying the zona pellucida and establishing a block to additional sperm entry (63). *In vitro* studies utilizing porcine models have demonstrated that prostasome-like vesicles can influence sperm, specifically by inducing the acrosome reaction (64). The capacitated sperm first binds to the zona pellucida through specific receptors, triggering the acrosome reaction. This exocytotic event releases proteases and hyaluronidases from the acrosome, which digest the zona matrix and facilitate sperm penetration. Following successful acrosome reaction, spermatozoa traverse the zona pellucida to reach the perivitelline space (PVS). Membrane fusion between sperm and oocyte plasma membrane is then enabled by capacitation-primed proteins, ultimately leading to fertilization. This process also promotes the targeted delivery of several regulatory substances to the female reproductive system, thus aiding fertilization (65).

The oocyte plasma membrane, prior to fertilization, expresses CD9 and CD81, which are crucial for the successful fusion of sperm and oocyte. CD9 is present on the sperm that achieves fertilization, playing a pivotal role in effective membrane fusion. CD9-positive EVs are detectable on the oocyte membrane, particularly on the microvilli where sperm attachment occurs. EVs enriched with CD9, released by the oocyte into the PVS, facilitate sperm-oocyte fusion by transferring CD9 to the sperm membrane. CD9-deficient show abnormalities in their microvilli and are unable to fuse with sperm (66). Oocytes lacking CD9 were fertilized with polemical material containing CD9 tetramer; however, another study reported that fertilization capacity of these oocytes lacking CD9 could not be rescued (67, 68). A different tetramer, CD81, is mainly synthesized by cumulus cells and is mostly found in the inner region of the zona pellucida, where it could play a role in fertilization, especially during pre-fusion processes like the acrosome reaction (66). As sperm penetrate the PVS, CD9 and CD81 can be transferred to the sperm via EVs (66). Abnormal expression of proteins of these proteins can negatively impact sperm function and fertilization.

EVs from the fallopian tube in mice have been shown to transfer miRNAs to sperm. For example, miR-34c-5p within oviductal EVs is transferred to sperm and is essential for initiating the first cleavage during fertilization (69). Hsa-miR-92a and hsa-miR-130b exhibit elevated expression levels in unfertilized follicular fluid, suggesting their potential as biomarkers for fertilization (70). However, the precise regulatory mechanisms warrant further investigation.

## 5 EVs role in embryo development and implantation

Pregnancy initiation requires a coordinated progression between the embryo and the endometrium, involving interactions throughout both the pre-implantation stage and the subsequent placental development. Various secretory factors from the endometrium have been recognized in uterine fluid, which has the potential to affect embryo development, endometrial epithelium adhesion, and overall functionality during the implantation phase (71) (**Figure 4B**). As a result, communication between the embryo and the endometrial lining is essential for effective implantation. Studies have shown that EVs released by both the trophoblast cells and the endometrium are crucial in promoting intercellular interaction at the maternal-fetal junction in early pregnancy (72).

EVs derived from the female reproductive tract, such as fallopian tube epithelium, can alter embryonic transcript expression when introduced into embryo culture (73). The addition of seminal EVs to *in vitro* fertilization media enhances blastocyst formation rates, extends embryo viability, diminishes apoptosis in blastocysts, and elevates embryo quality in murine models (74). Incorporating tubal bodies sourced *in vivo* into *in vitro* culture systems enhances embryonic development and quality. Notably, EVs derived from bovine fallopian tube epithelial cells have been shown to improve embryonic development, quality, and cryotolerance *in vitro* (75). Additionally, EVs derived from fallopian tube cells have increased the efficiency of mouse embryo transfer by decreasing embryonic cell apoptosis and promoting superior embryonic cell differentiation (76). Moreover, embryos can uptake EVs derived from the fallopian tube and endometrium, while embryonic EVs may regulate the fallopian tube and uterine functions (77).

EVs in uterine fluid enhance the proliferation of endometrial endothelial cells at the implantation site and regulate the endometrium, thereby supporting embryonic implantation. EV miR-92b-3p, originating from porcine endometrial epithelial cells, is internalized by porcine trophectoderm cells, where it modulates proliferation and migration (78). In addition, EV miRNAs enhance endometrial epithelial cell adhesion and promote blastocyst trophectoderm invasiveness during embryo implantation (79, 80). Embryo attachment is regulated by endometrial secretion of EVs into the uterine cavity, while the embryo also secretes EVs during implantation. Embryo-derived EVs modulate endometrial morphology and gene expression, influencing embryo positioning and implantation invasiveness (81). EVs from both embryonic and endometrial origins facilitate immune tolerance through crosstalk with the maternal system, thereby promoting implantation and pregnancy maintenance (82). miRNAs within EVs in peripheral blood may serve as valuable biomarkers for evaluating embryonic implantation (83). EV miRNAs, including miR-150-5p, miR-150-3p, miR-146b-3p, and miR-342-3p, are implicated in embryonic implantation and recognized as biomarkers for this process (84). The EV miR-17 and miR-20a, members of the miR-17/92 family, modulate trophoblast invasion and embryonic implantation by targeting TGF receptor II, Smad2, and Smad4, while both the miR-17/92 cluster and miR-1290 hold promise as novel biomarkers for evaluating endometrial receptivity and implantation potential (85–87). In addition, it has been demonstrated that trophoblast-derived EV miR-1290 can suppress the expression of LHX6, thus facilitating epithelial-mesenchymal transition and improving endometrial receptivity (87). EV miR-26b and miR-98 from the uterus downregulate maternal immune responses, facilitating conception and implantation (88). miR-30d and miR-200c within EVs derived from endometrium are implicated in embryonic implantation by modulating gene expression (89). Additionally, conceptus-derived EVs containing interferon-tau modulate gene expression associated with implantation, promoting progesterone synthesis and facilitating pregnancy establishment (90–92).

Research shows uterine fluid EVs regulate endometrial function and embryo implantation. In pregnant sheep, these EVs carry endogenous beta retroviruses env and gag RNAs that are transferred between trophoblast and endometrial cells, promoting embryonic trophectoderm development and placental expansion through cellular proliferation and tissue remodeling (93). EVs secreted by the endometrial epithelium are pivotal in mediating miRNA and adhesion signals to the blastocyst and the surrounding endometrium, thereby influencing endometrial receptivity and embryonic implantation (94). These findings offer new insights into the physiological significance of EV secretion of genomic information.

EVs can function autonomously, yet they often engage in synergistic interactions with soluble growth factors and hormones, highlighting their complex role in intercellular signaling (95). Analyses utilizing bioinformatics suggest that miRNAs specific to EVs can target biological pathways closely associated with the process of embryonic implantation (48, 96). Therefore, EVs that harbor particular miRNAs are found within the microenvironment of embryonic implantation and might significantly influence the relationship between the embryo and the endometrium (97). Endometrial-derived EVs containing miRNAs regulate embryo implantation in mice by enhancing levels trophoblast cell adhesion. In summary, EVs originating from the fallopian tube and endometrium lining promote interaction between the embryo and the maternal system during pregnancy (98).

## 6 EVs in reproductive diseases

**Figure 5:**
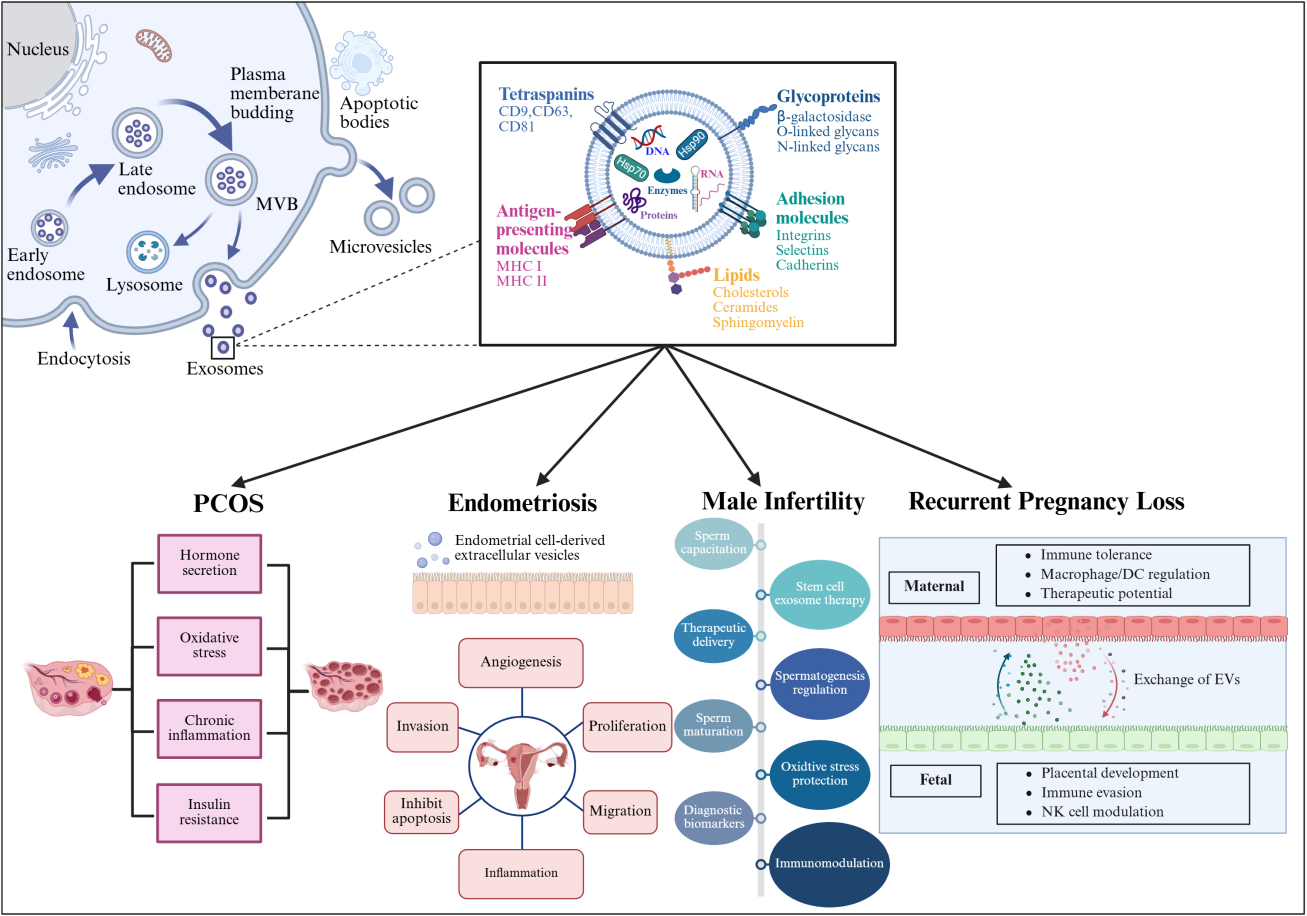
This figure illustrates the process of EV generation and their contents, including proteins, RNA, and lipids. The lower part of the image specifically details the role of EVs in four reproductive system-related diseases: Polycystic Ovary Syndrome (PCOS), endometriosis, male infertility, and recurrent pregnancy loss. In these conditions, EVs influence cellular communication by carrying specific biomolecules, thereby contributing to disease progression and pathogenesis. Created with BioRender.com.

In the past few years, EVs have attracted growing interest concerning their function in reproductive system diseases, including PCOS, endometriosis, male infertility, and RPL. The subsequent sections will discuss the role of EVs in diverse reproductive disorders, with a specific emphasis on their involvement in the immune system (**Figure 5**).

### 6.1 PCOS

EVs exert multifaceted regulatory roles in polycystic ovary syndrome (PCOS) pathophysiology by delivering specific miRNAs that orchestrate critical molecular pathways. These vesicles modulate energy supply through miR-34a-5p, which suppresses lactate dehydrogenase A to impair glycolysis in granulosa cells, while miR-143-3p disrupts Smad1/5/8 signaling pathway by targeting BMPR1A, promoting granulosa cell apoptosis (99, 100). Within the ovarian microenvironment, miR-379 inhibits granulosa cell proliferation via phosphoinositide-dependent kinase 1 upregulation and impedes M2 macrophage polarization, contributing to follicular developmental arrest (20). In PCOS mouse model, serum-derived miR-128-3p was down-regulated, which promotes granulosa cells ferroptosis (101). Notably, mesenchymal stem cell-derived EVs (e.g., BMSCs-Exo) counteract ovarian dysfunction by delivering therapeutic miRNAs that attenuate CD31 overexpression, normalize aberrant angiogenesis, and inhibit NF-κB-mediated inflammation in granulosa cells (102). Clinically, circulating EV miRNAs such as miR-143-5p and miR-34a-5p exhibit strong correlations with gamma-linolenic acid, serving as dynamic biomarkers for inflammatory monitoring in PCOS (103). EVs serve as effective drug delivery carriers, enabling the targeted delivery of therapeutic agents to specific cells. For instance, the delivery of anti-inflammatory drugs and insulin sensitizers through EVs can significantly ameliorate the inflammatory state and enhance insulin sensitivity in patients with PCOS, ultimately leading to improved treatment outcomes (104–106). Collectively, these insights underscore EV miRNAs as central mediators in PCOS pathogenesis, offering a dual promise for precision diagnostics and targeted therapies.

### 6.2 Endometriosis

Endometriosis is a common gynecological condition marked by the presence of endometrial tissue situated beyond the uterus. EVs are crucial in the pathophysiological mechanisms linked to endometriosis. Specifically, EVs containing miRNAs and growth factors facilitate cell migration and invasion. For instance, endometrial cells transfer miR-15a-5p through EVs, which ultimately contributes to the development of ectopic lesions (107). Furthermore, lncRNA carried by EVs, such as CHL1-AS1, can inhibit cell migration and proliferation (108). Additionally, EVs carrying IL-10 can inhibit NK cell activity, further facilitating the development of ectopic lesion (109). EV protein markers in blood can be leveraged for early diagnosis and monitoring of endometriosis. Research has shown that the expression levels of miR-22-3p and miR-320a in peripheral blood are positively correlated with the severity of endometriosis, establishing their potential as reliable biomarkers for the condition (110). Lastly, EV-mediated gene therapy may represent a novel therapeutic strategy. EVs demonstrate significant therapeutic potential for endometriosis management, as their capacity to transport bioactive cargo to designated cellular or tissue targets enables their employment as precise drug delivery vehicles and instruments for targeted therapeutic interventions (21).

### 6.3 Male infertility

Male infertility is a multifaceted condition influenced by numerous factors related to spermatogenesis and sperm maturation. Testicular sertoli cells and germ cells transfer miRNAs and proteins through EVs, influencing the development of male germ cells. For instance, miR-34b is transferred via EVs and regulates the sperm motility and count (111). In addition, EVs that carry antioxidant enzymes help regulate oxidative stress within sperm cells, thereby protecting them from damage (112). EVs originating from Sertoli cells have demonstrated the ability to prevent spermatogonial stem cell apoptosis by transferring miRNAs like miR-10b, resulting in the downregulation of KLF4 expression (113). Mesenchymal Stem-Cell derived EVs can contribute to attenuating cell injuries through specific miRNAs, such as miR-19a, miR-21-5p, and miR-144 (114). The possible role of these miRNAs in alleviating sperm damage caused by chlamydia indicates potential therapeutic use for EVs. Furthermore, the detection of EV miRNAs in semen may serve as valuable biomarkers for male infertility. It has demonstrated that semen miRNA levels correlate positively with sperm quality and male fertility, underscoring their potential as biomarkers for assessing these reproductive parameters (22). In treatment, EV delivery of antioxidants or miRNAs can ameliorate the oxidative stress in sperm, consequently enhancing sperm quality and fertility. Administration of superoxide dismutase (SOD) or miR-126-5p via EVs has been shown to maintain sperm viability and morphology, ultimately contributing to improved male fertility (115, 116).

### 6.4 Recurrent pregnancy loss

Recurrent pregnancy loss (RPL) is a prevalent complication during pregnancy characterized by a complex pathogenesis, in which immune factors play a pivotal role. In recent years, the immune regulatory functions of EVs in RPL have garnered significant attention. miRNAs, proteins, and lipids carried by EVs, which are essential for maintaining immune balance at the maternal-fetal interface (117).

Decidua-derived EVs have been shown to be essential modulators of T-cell differentiation and function through the delivery of specific miRNAs, thereby facilitating the development of immune tolerance (118, 119). This immunoregulatory mechanism extends to their capacity to activate macrophages and dendritic cells while maintaining inflammatory homeostasis - a critical function for protecting the embryo from pathological immune response (120). Stem cell-derived EVs have demonstrated notable immunosuppressive and anti-inflammatory properties. A landmark study by Xiang et al. employed ultracentrifugation to isolate EVs from bone marrow mesenchymal stem cell cultures, which were then administered to pregnant mice with a history of RPL. The intervention resulted in significantly improved pregnancy outcomes, as evidenced by increased serum levels of the anti-inflammatory cytokines IL-4 and IL-10, along with concomitant reductions in proinflammatory mediators TNF-α and IFN-γ at the maternal-fetal interface. Mechanistically, this therapeutic approach modulated both T-cell function and macrophage polarization, ultimately decreasing embryo resorption rates by 42% compared to control groups. These findings collectively establish the therapeutic potential of stem cell-derived EVs in ameliorating immune-mediated pregnancy complications through precise immune modulation (121). Another investigation showed that villi can modulate IFN-γ production by decidual natural killer cells via the EV-mediated delivery of miR-29a-3p. This finding suggests a novel therapeutic strategy involving engineered villus-derived EVs mixed with HA-Gel, which shows promise for treating unexplained RPL in both murine models and potential clinical applications (23). Collectively, the immune regulatory role of EVs offers a novel perspective for understanding pathogenesis of RPL. By exploring the specific mechanisms of EV-mediated immune regulation at the maternal-fetal interface, we may identify new targets and strategies for the early diagnosis and treatment of RPL (122). Nonetheless, the prospect of utilizing EV therapy for managing RPL appears encouraging, especially when combined with current therapeutic strategies.

## 7 Conclusions and future directions

**Figure 6:**
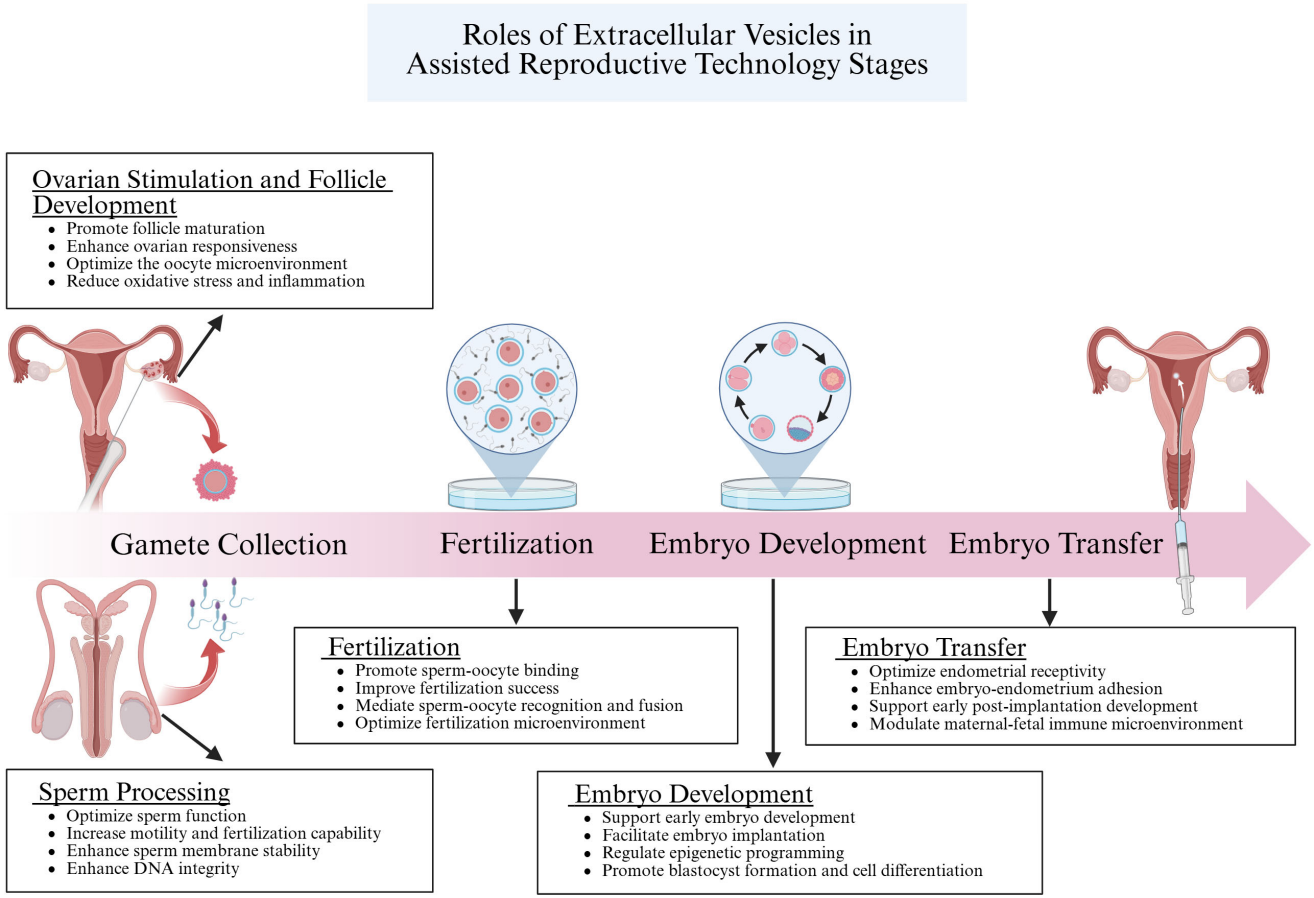
This figure elucidates the crucial roles of EVs in Assisted Reproductive Technology (ART). EVs enhance the quality of sperm and oocytes by regulating the microenvironment of the reproductive tract and delivering signaling molecules and bioactive substances. During fertilization, they facilitate the recognition and binding between sperm and oocyte by transferring specific proteins and molecules, thereby increasing the success rate of fertilization. In the embryo development stage, EVs are vital in regulating gene expression and cell differentiation through intercellular communication, ensuring proper embryonic growth. Finally, during embryo transfer, EVs support the preparation of the uterine endometrium and enhance embryo-uterus interactions, which improves the implantation potential of the embryo. Collectively, these processes demonstrate the significant impact of EVs on the success of ART. Created with BioRender.com.

In conclusion, EVs play a role in numerous biological functions in reproductive systems, such as gamete maturation, fertilization, embryo development, and the progression of reproductive diseases. These small vehicles carry bioactive compounds such as proteins, lipids, and nucleic acids from one cell to another, functioning as crucial regulator of cell communication. Their potential as indicators and therapeutic targets is highlighted by their involvement in these vital reproductive processes. EVs aid in the interchange of vital components that improve sperm and oocyte quality during gamete maturation, increasing the chance of successful fertilization (**Figure 6**). EVs regulate gene expression and cellular signaling during embryogenesis, fostering proper embryonic development and differentiation. Their dysregulation is associated with reproductive disorders, highlighting the importance of understanding their mechanisms. For ART, the use of EVs is especially promising. Clinicians can utilize EVs to develop innovative diagnostic tools and therapeutic strategies to combat infertility and enhance ART outcomes. EV-based interventions could enhance the quality of gametes and embryos, reduce the risk of implantation failure, and minimize the incidence of pregnancy complications.

Future research should focus on elucidating the specific molecular pathways and cargo of EVs involved in reproductive processes, while also exploring their potential applications in personalized medicine. Integrating EV into clinical practice has the potential to revolutionize the reproductive medicine, providing new hope to couples facing infertility diseases. In sum, research into EVs within reproductive biology and pathology deepens our comprehension of human reproduction. With ongoing advancements, the significance of EVs is anticipated to escalate, paving the way for innovative strategies in reproductive health.
